# RACE BY AGE PATTERNS IN KIDNEY FUNCTIONING AMONG OLDER ADULTS: EVIDENCE FROM THE HEALTH AND RETIREMENT STUDY

**DOI:** 10.1093/geroni/igz038.1284

**Published:** 2019-11-08

**Authors:** Ryon J Cobb

**Affiliations:** University of Texas at Arlington, Arlington, Texas, United States

## Abstract

The present study considers how race combines with chronological age to shape kidney function among older adults. We analyzed cross-sectional data from a nationally representative study of older adults. Our measure of kidney function derived from the cystatin C-based estimated glomerular filtration rate. We use a pattern variable to divide White and Black respondents into four groups based on their age group membership: early midlife (age 52–59), late midlife (age 60–69), young old (age 70–79), and oldest old (80s+ years). Results from our ordinary least squares models reveal that Blacks and Whites in late midlife, young old, and oldest old exhibited poorer kidney function than Whites in early midlife. Our study uncovers evidence of race by age disparities in kidney function among older adults. Future longitudinal studies will provide further insight into how and why race combines with age to pattern kidney function over time.

